# An experimental study on grammatical sensitivity and production competence in Chinese and Spanish EFL learners and its implications on EFL teaching methods

**DOI:** 10.3389/fpsyg.2023.1096875

**Published:** 2023-02-21

**Authors:** Qiaoling He, Isabel Oltra-Massuet

**Affiliations:** ^1^College of General Education, Sichuan International Studies University, Chongqing, China; ^2^Department of English and German Studies, Universitat Rovira i Virgili, Tarragona, Spain

**Keywords:** EFL learners, implicit knowledge, grammatical sensitivity, language production competence, interaction-based POA tasks

## Abstract

Implicit knowledge acquired by L2 learners determines their language competence; however, it remains an issue to what extent advanced EFL learners can acquire implicit language knowledge. This study aims at finding out whether advanced EFL learners from two different L1s could acquire a level of implicit knowledge of English questions by using the modified Elicited Oral Imitation Task. A quantitative, experimental study with the Elicited Oral Imitation Task experimental tool was designed. A total number of 91 participants were recruited *via* the online experimental platform from October to November, 2021, distributed into a native speaker group, a Chinese EFL learner group, and a Spanish EFL group. The study evaluated participants’ implicit language knowledge by assessing two indicators: the grammatical sensitivity index and the production index. Independent-sample *t*-test and one-way analysis of variance (ANOVA) were applied to examine the differences in the two indices among different groups. Results revealed that both EFL groups displayed a significant difference with the native speaker group in their degree of implicit knowledge of English questions in general. A further comparison of the two indicators showed that while both EFL groups displayed a relatively high grammatical sensitivity to morpho-syntactic errors in English questions, their corrective production rate of ungrammatical sentences was notably lower. These results indicate that advanced EFL learners had difficulty in acquiring implicit knowledge of English questions at native speaker’ level. These findings also imply a gap between EFL learners’ language knowledge level and corresponding language production competence. Targeting this gap within the Interaction-based production-oriented approach pedagogical implications based on were suggested for enhancing EFL learners’ language production competence in EFL contexts.

## Introduction

The extent to which second language (L2) learners can acquire implicit knowledge (IK) largely determines their language competence, including comprehension and production (Paradis, 2009), but whether L2 learners can acquire IK has remained a matter of debate (VanPatten et al., 2020). It is not unusual for researchers and practitioners to see that advanced English as a Foreign Language (EFL) learners who can articulate grammar rules and perform excellently in various written examinations still produce ungrammatical morpho-syntactic structures in sentences that appear to be simple in spontaneous oral communication. This kind of ‘what I know’ and ‘what I can’ incongruity aroused our interest to experimentally probe whether EFL learners’ language knowledge and language production competence develop simultaneously as that of native speakers. A number of previous studies have investigated L2 learners’ implicit language knowledge by testing their grammatical sensitivity (Tokowicz and MacWhinney, 2005; Abrahamsson and Hyltenstam, 2008; Roberts and Siyanova-Chanturia, 2013; Zhang, 2015; Suzuki and DeKeyser, 2017), whereas little research has studied learners’ language production, despite its importance in evaluating learners’ L2 acquisition (Kowal and Swain, 1994; Grüter et al., 2012; VanPatten and Williams, 2015).

Aiming at finding out whether and to what extent advanced EFL learners can acquire L2 IK, the present study first addresses the issue of IK acquisition by comparing advanced Chinese and Spanish EFL learners’ imitated production of English questions with native speakers, with an experiment subsuming a consecutive process of grammatical sensitivity and language production. The second objective of this study is to delve into the language acquisition trajectory for EFL learners. We tackle the development of language knowledge and language production competence through the analysis of the experimental data unveiling learners’ grammatical sensitivity and production competence. Finally, building on the experimental findings, the study puts forward a series of pedagogical implications for course designers and class practitioners in EFL contexts, targeting at enhancing learners’ language production competence.

The following two research questions are formulated to address the first and second goals, the IK acquisition and the development of grammar knowledge and production competence.

*RQ1*: Do advanced EFL learners from different L1s acquire native-equivalent IK in English questions?

*RQ2*: Does advanced EFL learners’ language production competence develop simultaneously with their grammatical knowledge of English questions?

## Background

### Implicit knowledge and its measurements

The concept of implicit knowledge is elusive and has proved difficult to confront against the notion of general knowledge learning. To address this issue, Berry (1987) distinguished two types of IK, the IK that was once explicit and declarative and the IK that arose from implicit learning, which had never been explicit. In the domain of language acquisition, the former kind of IK defined by Berry explains the L2 learning process in EFL learners, while the latter accounts for the First Language (L1) acquisition process in native speakers. In L2 acquisition, Ellis and Roever (2018) defined implicit language knowledge as the knowledge that learners have not consciously noticed but can access in spontaneous language production through automatic processing, even if it cannot be verbalized. Ellis (2005) summarized the key characteristics of IK through seven properties: intuitive awareness, procedural knowledge, systematicity, automatic processing, accessibility in fluency performance, non-verbalizable language rules, and learnability.

Among all the seven characteristics categorized by Ellis (2005), learners’ awareness of grammar has been the focus in studies of implicit language knowledge measurement (Spada et al., 2015; Zhang, 2015; Kim and Nam, 2016; Suzuki and DeKeyser, 2017; Roehr-Brackin, 2020). To measure learners’ IK, some studies have tested learners’ grammatical awareness in the process of language comprehension using tools such as timed grammatical judgment, word monitoring test, or self-paced reading, *i.a.* (Gutiérrez, 2013; Suzuki, 2015; Suzuki and DeKeyser, 2017). Other research has evaluated learners’ grammar awareness in the process of language production employing tools such as elicited imitation or oral narrative (Ellis, 2005; Erlam, 2006; Spada et al., 2015; Zhang, 2015; Suzuki and DeKeyser, 2017). These previous findings laid a sound empirical foundation for further experimental studies on implicit knowledge. However, existing studies, by either collecting data in language comprehension or language production, only took learners’ awareness as the indicator for evaluating learners’ implicit knowledge, without paying much attention to learners’ production, although production is regarded as essential for explaining the language acquisition process (Kowal and Swain, 1994; McDonough and Chaikitmongkol, 2010; Grüter et al., 2012; Guasti et al., 2012; MacDonald, 2013). Considering the role language production plays in language acquisition, the present study aims at focusing on both learners’ awareness of grammar (grammar sensitivity) and competence of production (corrective language production) to explore implicit language knowledge taking the acquisition of English questions by EFL learners as empirical target basis. The study will evaluate the level of advanced EFL learners’ implicit knowledge through these two perspectives and explore whether advanced EFL learners’ competence of production develops simultaneously with their grammar knowledge in English questions.

### Grammatical sensitivity

Grammatical sensitivity, according to Sasaki (2012), was defined as learners’ ability to identify the grammatical role of certain words or sentence components in given sentence structures. Students with grammatical sensitivity were able to detect relationships between words and their grammatical function in the sentence (Vanpatten et al., 2013). In Sasaki (2012) and Vanpatten et al. (2013), grammatical sensitivity was defined as learners’ ability to recognize the grammatical roles of sentence components, but in more recent studies (Tokowicz and MacWhinney, 2005; Abrahamsson and Hyltenstam, 2008; Keating, 2009; Roberts and Siyanova-Chanturia, 2013), grammatical sensitivity refers to the sensitivity degree that learners show to grammatical violations in ungrammatical structures. The event-related brain potentials (ERPs) data from Tokowicz and MacWhinney (2005) suggested that L2 learners across different proficiency levels were implicitly sensitive to grammatical violations. Keating (2009) found that grammatical sensitivity displayed by adult L2 Spanish learners can be a robust predictor of their aptitude to acquire the structure of gender agreement. Abrahamsson and Hyltenstam (2008) suggested that late learners’ high grammatical sensitivity indicated their high language proficiency. Roberts and Siyanova-Chanturia (2013) supported that assessing learners’ sensitivity to ungrammatical sentence structures in processing comprehension can uncover how that acquired language knowledge is used in real-time language processing.

These studies revealed that sensitivity to grammatical violation is an important indicator in assessing L2 learners’ IK acquisition (Suzuki, 2017; Vafaee et al., 2017). The present study adopted the concept of grammatical sensitivity in terms of learners’ reactions to grammatical violations to evaluate EFL learners’ level of implicit knowledge in acquiring morpho-syntactic structures of English questions. Therefore, grammatical sensitivity in this study is exclusively defined as the learners’ capacity to recognize the grammar components in English questions tacitly and to unconsciously display a delay in reaction to the ungrammatical features in English questions under experiment. The grammatical sensitivity index, referring to the percentage of ungrammatical sentences detected by participants, is used to quantify learners’ sensitivity levels.

### Language production

Language production, together with language comprehension, constitutes an interwoven process in the development of language competence when language learners receive input and create output. Recent research done by cognitive psycholinguists such as Pickering and Garrod (2007) regarded that language comprehension and production came from the same system, and the production system was used when prediction and imitation were activated to emulate how imitation and comprehension worked. According to Krashen’s (1982) monitor theory, accurate and fluent language production is initiated with the acquired system of knowledge. Language production promoted learners’ language learning by helping learners become aware of their existing grammar knowledge gap and enhance their awareness of the links between forms, function, and meaning, which played an important role in L2 acquisition (Kowal and Swain, 1994). Language production reveals learners’ real-time processing of language structure, which provides important data for analysing learners’ persistent difficulty in acquiring specific language structures (Grüter et al., 2012). As an additional indicator for learners’ acquisition of linguistic knowledge, L2 learners’ production offers an approach to studying the degree of knowledge acquired by L2 learners. However, so far, not much research focusing on production has been done because of the difficulty in designing an appropriate task to measure learners’ language competence (VanPatten and Williams, 2015). In particular, it is not feasible to capture language data in natural language to study a specific target structure. The present study conducts a production experiment to overcome the issue raised by Gass and Mackey (2015) that participants may avoid producing the target structures, eliciting participants to generate the structures of interest with stimuli sentences. We specifically defined learners’ production as the number of sentences participants produced with given stimuli. In the elicitation process, participants noticed the ungrammatical features and made corresponding grammatical sentences. We use the production index to designate the percentage of participants’ corrective production of ungrammatical experimental sentences.

### The oral elicited imitation test

The study chose the elicited oral production test to collect data for measuring EFL learners’ IK from language production among the three major categories of experimental methods: (1) a battery of tests including timed grammatical judgment, oral elicited imitation test (OEIT), and oral narrative designed by Ellis (2005); (2) a series of reaction time (RT) tests (Suzuki, 2015) covering visual word paradigm, word monitoring test, and self-paced reading; and (3) a set of cognitive neurolinguistic tools comprising eye-tracking (Keating, 2009; Conklin and Pellicer-Sánchez, 2016; Maie and Godfroid, 2022), event-related potentials (ERPs) (Tokowicz and MacWhinney, 2005; Dowens et al., 2011; Martínez de la Hidalga et al., 2021), and functional magnetic resonance imaging (fMRI) (Xue et al., 2004; Yokoyama et al., 2006). The OEIT has been considered a promising option since in RT tests and cognitive neurolinguistic experiments, participants were tested mainly in comprehension, not involving learner production, which leaves the question of whether there is a gap between learners’ sensitivity and production unsolved.

The OEIT originated from elicited imitation (EI) that can be dated back as early as the 1970s. Early researchers, such as Crain and Thornton (1983), had designed elicited production to perform empirical studies of learners’ language competence. However, there has been constant questioning over its effectiveness. Vinther (2002) reviewed studies on the application of EI in child language, neuropsychology, and second language research from 1970 to 1994 and suggested that EI was able to test learners’ process of language in comprehension and production under the condition that it was applied with careful consideration of variables such as imitation process, the stimulus length and structure, and linguistic contextual support. On the state that stimuli sentence items of target grammatical features are well-designed, EI could test both learners’ sensitivity to knowledge in tacit forms as well as learners’ competence in production directly. In the present study, we modified the experimental stimuli sentences by fully considering variables such as sentence length, structure, and contextual support to ensure effective measurement of learners’ grammar sensitivity as well as their production.

The OEIT caters best to the research goals of the present study in the sense that it incorporates experimental procedures testing participants’ tacit grammatical judgment and direct corrective production. With OEIT experimental data, the authors are able to dissociate participants’ performance into grammatical sensitivity and language production indexes. Moreover, the OEIT has been regarded as an effective tool for measuring learners’ IK, with its measuring power for language learners’ knowledge having been replicated and validated in previous research (Bowles, 2011; Spada et al., 2015; Zhang, 2015; Kim and Nam, 2016). In contrast to earlier studies employing OEIT, the current study intends to go beyond validating and replicating the findings. We adopt the OEIT test tool in this study to collect data from participants’ production, intending to investigate the participants’ performance from the perspectives of both grammatical sensitivity and production competence.

As reviewed in this section, grammatical sensitivity and language production are two crucial indices for studying implicit knowledge, and OEIT can actually test both. Existing studies on implicit knowledge have mainly focused on analysing grammatical sensitivity, without paying much attention to the production index. In fact, previous OEIT studies have not focused on the production index, either. However, OEIT tasks tacitly tested participants’ sensitivity to grammatical violation and consecutively tested the production of experimental sentences, thus offering the possibility to study both sensitivity and production. In contrast to previous research, the present study analyses implicit knowledge acquired by EFL learners from the perspectives of both grammatical sensitivity and production competence and further investigates the development of grammar knowledge and production competence of participants.

## Methodology

### Research design

A quantitative study with the OEIT experimental tool is designed to reveal EFL learners’ degree of implicit knowledge and explore the relationship between the acquisition of language knowledge and language competence.

### Participants

The sample size is based on related studies in SLA (Ellis, 2005; Suzuki and Dekeyser, 2015; Suzuki and DeKeyser, 2017). All participants were recruited with simple random sampling *via* the experiment participants’ recruitment platform https://www.prolific.co/, which directs participants straight to the experiment platform.^1^ The study recruited a total number of 91 participants, but only received valid data from 84 participants, comprising monolingual English native speakers (*n* = 12), Chinese (*n* = 35), and Spanish (*n* = 37) learners of English, after removing those who did not correct any grammatical sentences or provided less than 75% correct answers to comprehension judgments. All native speaker participants are monolingual English speakers, with an education level of undergraduate or above. All EFL participants are advanced learners with an English proficiency level at or equivalent to the C1 level following the CEFR (*The Common European Framework of Reference for Languages*). We further qualified all participants by adding a short C1-level test, filtering out those who could not give three correct answers to five questions. The short C1-level test comes directly from the official Cambridge English test paper, so the test content is reliable for testing participants language proficiency. Before the experiment, we conducted a pilot test with the short C1-level with 8 EFL learners for validation. By comparing their test results with their reported C1-level scores, we got a Spearman correlation coefficient of 0.761 (*p* = 0.028), showing that the short C1-level test has high validity. Participants from both EFL groups are equivalent in their age, education, and starting time of English learning, guaranteeing an effective comparison of the experimental data. Participants were aged from 18 to 40, most of whom were in the age range 21–40 years old. Over 90% of participants were undergraduates or graduates, and most of them started to learn English in primary or secondary school (Supplementary Material shows detailed demographic information of participants).

### Experimental tool

Aimed at testing participants’ grammatical sensitivity and corresponding production ability, we created a modified experimental tool based on the OEIT (Ellis, 2005). The test in the present study adopted essential criteria for operationalizing constructs from Ellis (2005): intuitive, time–pressure, meaning-focused, and consistent responses without relying on explicit grammatical rules.

The OEIT was updated and revised in the following aspects. First, we added pictures to test participants’ understanding of the content of experiment sentences. Linguistic structures tested in OEIT in previous studies were mainly statements, allowing participants to make immediate True/False judgments right after listening to sentence prompts without extra incentives. But in this study, all experimental sentences are questions, with equal amount of grammatical and ungrammatical statements included as fillers to disguise our experimental aim on questions. It is thus impossible for participants to make meaning-focused True/False judgments directly. We presented pictures on the screen simultaneously with the recording to test participants’ comprehension of experimental sentences. Second, two improvements discussed in previous validation research (Spada et al., 2015; Kim and Nam, 2016) were also incorporated into the present test. Since truth-value judgment (used in Spada et al., 2015) is based on the content of the given sentences and is more objective than the belief statement, the current test chose to use truth-value judgments to ensure that learners have processed the sentence stimuli for meaning. True/False judgment was designed in choice (A/B) to test participants’ understanding of the content; furthermore, a time limit was added to the test, which allowed participants 20% more time than native speakers (used in Kim and Nam, 2017).

After we designed the initial experimental sentence items, two native speakers were invited to check the naturalness, understandability, and grammaticity of each grammatical item to confirm the face validity of the items. Based on their feedback, we revised all points they provided with correction feedback. Furthermore, to testify the experimental validity and responsibility of the modified OEIT, we conducted a pilot study with 8 native speakers. The results of the pilot study showed a 100% response rate, with an averaged accuracy of 96.9% in grammatical sensitivity, and 96.6% in production competence, suggesting that this modified OEIT effectively measured the level of implicit knowledge.

### Experimental procedures

**Figure 1:**
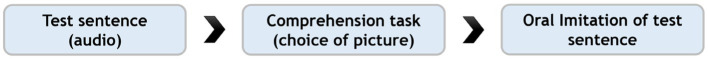
Experimental procedures of Elicited Oral Imitation Task.

The experiment was approved by the Research-Innovation Ethics Committee from Universitat Rovira i Virgili, Tarragona, Spain. The whole experiment was conducted in the online platform www.Gorilla.sc. After participants read the experimental instruction and ticked the consent form, they filled out a questionnaire about their language learning background. Participants were automatically directed to the experiment page after passing a short proficiency test. The instructions were displayed on the screen with words, pictures, and audios. They informed participants that they might hear a grammatical or an ungrammatical sentence. While the audio (the stimulus) was played, participants had to press a key as a response when they heard the target word that was displayed on the screen. The target word was presented simultaneously to the entire duration time of the audio stimulus. Participants needed to respond quickly by pressing the key before the experimental webpage automatically switched to the next screen. Participants’ RT for measuring sensitivity was automatically recorded by the online experimental platform when the key was pressed. To further confirm the authenticity of the RT, pictures (including one picture that revealed the situation/content and one unrelated picture) were used to check whether participants understood the meaning of the stimuli sentences. They needed to choose the picture that matched the content of each sentence. Next, participants were guided to repeat the sentences orally using correct English, and their productions were audio recorded. After the instruction, they completed five practical trials and could choose to re-practice if necessary. All their comprehension answers and their utterances were recorded as experimental data. The experimental procedure is shown in Figure 1.

### Scoring

Before scoring, filler sentences and invalid experimental sentences with incorrect comprehension judgments were eliminated, and we only kept the critical experimental sentences with correct comprehension judgments. The scoring criterion was based on obligatory occasions (Dulay and Burt, 1973). In this case, only matched question structures produced by participants were included in the analysis because participants needed to form the same type of question structure as what they heard in the audio. Each participant’s grammatical sensitivity and production accuracy score was marked separately. One score was given for a correct response and zero for an incorrect one, with a total score ranging from 0 to 40. A higher score indicated a higher sensitivity or production accuracy. The scoring was independently done by one researcher and a research assistant, and the score of each item was double checked. Any disagreement in scores was solved after discussion. The scoring criteria in terms of sensitivity and production score for ungrammatical and grammatical experimental items are specified in Supplementary Material.

### Statistical analysis

Descriptive statistics, including frequency and constituent ratio, were used to describe participants’ demographic characteristics and the overall performance of each group in grammatical sensitivity and language production. The Kolmogorov–Smirnov test suggested that the grammatical sensitivity and language production were not normally distributed; however, we selected parametric tests for data analysis due to the following two reasons: (1) the Kolmogorov–Smirnov test results flux and are not always reliable as the sample size varies, especially with a big sample size (Steinskog et al., 2007); and (2) normal distribution of these two indicators was assumed in the present study based on the Histogram, the Normal Q-Q Plot, and values of skewness and kurtosis (Ho and Yu, 2015). Therefore, we summarized grammatical sensitivity and language production using mean and standard error (SE). Meanwhile, independent-sample t-test and one-way analysis of variance (ANOVA) were applied to examine the difference in grammatical sensitivity and language production among different groups. When a statistically significant result was detected for the overall test using ANOVA, a post-hoc test was performed using Fisher’s Least Significant Difference (LSD) to investigate which group differed from the others in terms of grammatical sensitivity and language production. All statistical analyses were performed by using IBM Statistical Package for Social Sciences (SPSS) version 22.0 (SPSS Inc., Chicago, United States). A two-tailed *p* < 0.05 indicated statistical significance.

## Results

### Descriptive data

The participants produced 3,360 valid utterances. Among these sentences, 480 were from native speakers, 1,480 were from Spanish EFL learners, and 1,400 were from Chinese EFL learners. Native speakers showed grammatical sensitivity in 470 sentences (97.9%) and produced 468 sentences correctly (97.5%), indicating they are extremely sensitive to grammatical errors and highly proficient in correcting grammatical errors automatically. In comparison to the Spanish group, which produced 1,295 (87.5%) and 1,224 (82.7%) correct sentences out of 1,480 total, the Chinese EFL group demonstrated grammatical sensitivity at 1186 (about 84.7%) and produced 1,102 (78.7%) correct sentences.

The results showed that EFL learners developed a high sensitivity to ungrammatical structures, but their production lagged. To explore the relationship between sensitivity and production competence, we looked into the data of the native speaker group and the two EFL learner groups. We observed that native speakers corrected about 99.5% (468 out of 470) of ungrammatical sentences to which they showed grammatical sensitivity, i.e., they produced almost all ungrammatical sentences in correct forms. The results indicated a high degree of IK in their native language. For Chinese and Spanish learners, they successfully corrected 92.9% (1,102 out of 1,186) and 94.5% (1,224 out of 1,295) of the sentences where they detected grammatical errors (Table 1). The results revealed that advanced EFL learners could correct most ungrammatical errors they recognized, but their correction rates were much lower.

**Table 1:** Effective responses in grammatical sensitivity and production.

	Sensitivity N (%)	Production N (%)	Production-Sensitivity N (%)
NS group	470 (97.9%)	468 (97.5%)	468 of 470 (99.5%)
CH group	1,186 (84.7%)	1,102 (78.7%)	1,102 out of 1,186 (92.9%)
SP group	1,295 (87.5%)	1,224 (82.7%)	1,224 out of 1,295 (94.5%)

NS, native speaker; CH, Chinese speaker; SP, Spanish speaker.

### Results of between-group difference in sensitivity and production

Results of ANOVA indicated a significant difference in the overall score of sensitivity (*F* = 9.59, *p* = 0.000) and production (*F* = 13.69, *p* = 0.000) among the three groups. Furthermore, the LSD post-*hoc* multiple comparison tests showed that the mean sensitivity and production scores for the native group were, respectively, 39.2 and 39 out of 40 in total, significantly higher than the Chinese group with a large effect size for sensitivity (MD = 5.28, SE = 1.21, *p* < 0.001, Cohen’s *d* = 0.822) and a medium effect size for production (MD = 7.51, SE = 1.44, *p* < 0.001, Cohen’s *d* = 0.691), and the Spanish group with a large effect size for sensitivity (MD = 4.12, SE = 1.20, *p* = 0.001, Cohen’s *d* = 0.637) and with a large effect size for production (MD = 5.92, SE = 1.43, *p* < 0.001, Cohen’s *d* = 0.535). However, the difference in sensitivity and production between the two EFL groups was not significant, with a small effect size (MD = −1.11, SE = 0.85, *p* = 0.195, Cohen’s *d* = 0.219) for sensitivity and a small effect size (MD = −1.60, SE = 1.015, *p* = 0.120, Cohen’s *d* = 0.183) for production (see Table 2). Even though the two advanced EFL groups were highly sensitive to ungrammatical structures and were able to produce grammatical sentences with an accuracy rate of about 80%, they were at significantly lower level of sensitivity and production compared to native speakers.

**Table 2:** Results of between-group multiple comparisons with LSD test.

Test type	Comparison	MD	SE	Value of *p*	95% CI	
					Lower limit	Upper limit
Sensitivity	NS vs. CH	5.2809	1.20854	0.000	2.8763	7.6856
	NS vs. SP	4.1666	1.20018	0.001	1.7787	6.5546
	CH vs. SP	−1.1142	0.85186	0.195	−2.8092	0.5807
Production	NS vs. CH	7.5142	1.43931	0.000	4.6505	10.3781
	NS vs. SP	5.9189	1.42935	0.000	3.0750	8.7629
	CH vs. SP	−1.5953	1.01452	0.120	−3.6139	0.4232

NS, native speaker; CH, Chinese speaker; SP, Spanish speaker; MD, mean difference; SE, standard error; CI, confidence interval.

### Results of within-group of sensitivity and production

**Figure 2:**
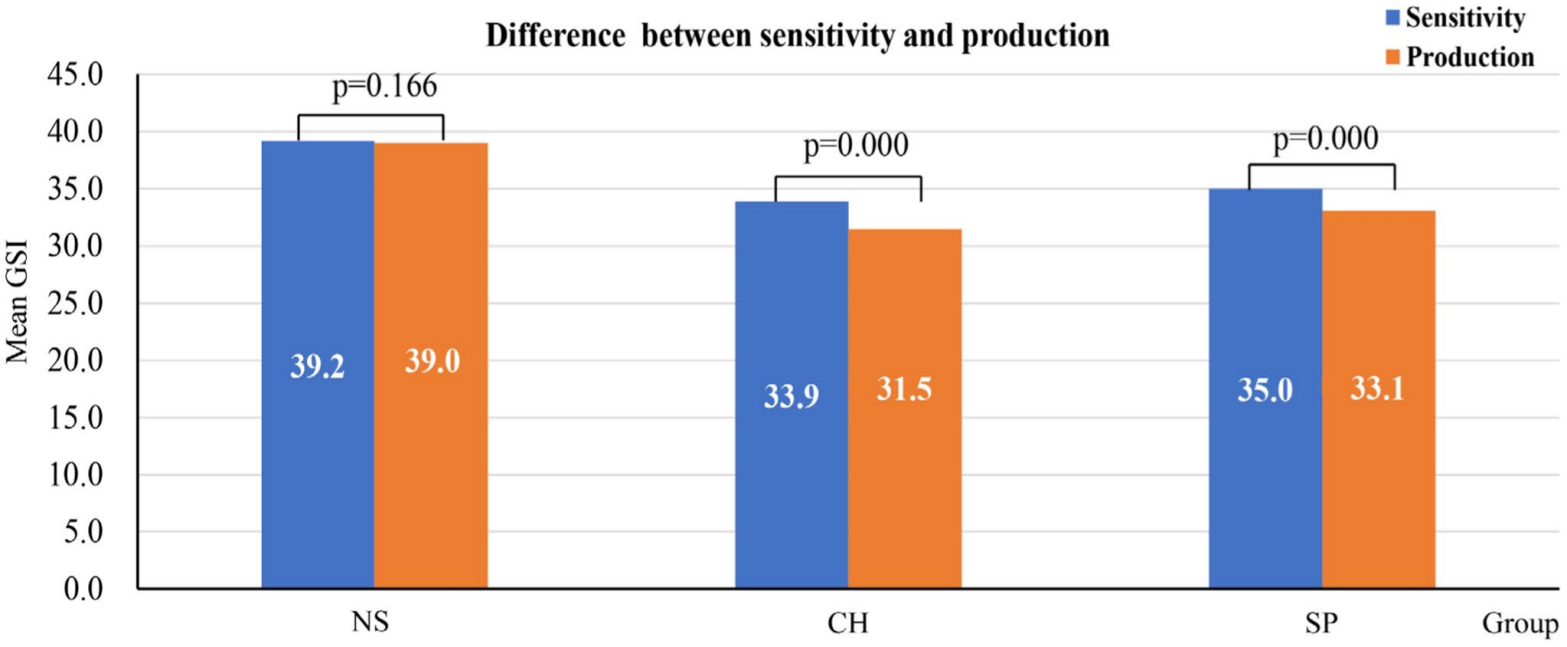
Paired *t*-test of difference between sensitivity and production. NS, native speaker; CH, Chinese speaker; SP, Spanish speaker.

We analysed participants’ sensitivity and production scores in order to further examine whether there was a notable gap between participants’ grammatical knowledge and production competence. The results showed no significant difference with a medium effect size (*t* = 1.483, *p* = 0.166, Cohen’s *d* = 0.605) in the NS group, but a significant difference in the Chinese with a large effect size (*t* = 7.364, *p* < 0.001, Cohen’s *d* = 1.760) and Spanish group with a large effect size (*t* = 8.703, *p* < 0.001, Cohen’s *d* = 2.023) (see Figure 2). The results demonstrated that native speakers possessed a high degree of implicit language knowledge that enabled them to produce correct sentences automatically. However, the results from Chinese and Spanish EFL learners revealed that, even at an advanced level, there was a notable difference between their language knowledge and language production competence.

## Discussion

The present study first shows that Spanish and Chinese advanced EFL learners have hardly acquired native speakers’ IK of English questions. The results also reveal that advanced EFL learners’ language production competence falls behind their language knowledge acquisition. This section will discuss EFL learners’ acquisition of English questions by analysing learners’ IK level and the development of language production competence from the perspectives of grammatical sensitivity and language production.

### Implicit knowledge of native speakers and advanced EFL learners

The results of both between-group and within-group comparisons answered the first research question. First, the native and EFL between-group comparisons showed that both Spanish and Chinese EFL learners’ sensitivity and production scores are significantly lower than Native speakers (as shown in Table 2), which suggests that EFL learners do not possess the same IK as native speakers. Therefore, we may conclude that it is difficult for EFL learners to acquire IK, even at an advanced proficiency level. As elaborated in the previous section, language learners who acquire IK are supposed to be highly sensitive to morpho-syntactic errors and capable of producing grammatical language with a high accuracy rate.

Another difference between EFL learners and native speakers comes from the comparison of their capacity for correcting morpho-syntactic errors to which they showed sensitivity. From the Production-Sensitivity data in Table 1, we can see that native speakers correct almost every error they detect, while the Chinese group and the Spanish group show a relatively lower correction rate. We assume that IK refers to an equivalency between sensitivity and production. Native speakers can make grammatical sentences automatically because their IK enables them to produce them as soon as they subconsciously recognize ungrammatical features. Therefore, for learners who have acquired IK, there should not be a significant gap between their grammatical sensitivity and production competence. However, the sensitivity-production gap reflected in both Chinese and Spanish groups is significant (shown in Figure 2), which suggests that the grammatical knowledge they acquired is not implicit. They did not show equivalent automatic correction to morpho-syntactic errors they recognized. These findings allow us to conclude that advanced EFL learners may have acquired some L2 IK but not IK *per se* of the native speakers.

Moreover, the native speaker group shows high consistency between their grammatical sensitivity and production, because their implicit L1 knowledge enables them to produce correct sentences as soon as they tacitly recognize the grammatical violation. It is sensible for us to propose that the consistency in the native group and inconsistency in the EFL groups stem from the difference in their degree of implicit language knowledge. As discussed above, native speakers could spontaneously produce norm-conforming language without relying on conscious grammar knowledge (Ellis and Roever, 2018). Native speakers’ L1 acquisition depends mainly on the IK they acquired *via* implicit learning, while EFL learners’ L2 acquisition generally comes from explicit and implicit knowledge through both explicit and implicit learning (Hulstijn, 2005). Native speakers and EFL learners acquire the language in distinct contexts and processes, which causes differences in the knowledge they acquire. We can surmise that advanced EFL learners’ differences in sensitivity and production stem from their lack of IK on par with their native counterparts.

### Grammatical sensitivity vs. production competence

Grammatical sensitivity reflects learners’ degree of acquired language knowledge, which subsequently influences their production competence (Vanpatten et al., 2013). Therefore, in this study, we studied advanced EFL learners’ grammatical knowledge level and language production competence to answer the second research question. We found that both Chinese and Spanish EFL learners show more robust competence in language sensitivity than in production, so we suggest that this inequivalence indicates that (1) advanced EFL learners’ grammatical knowledge develops prior to their production, and thus their acquisition process in EFL contexts tends to be more explicit, and that (2) advanced EFL learners still confront difficulty in accuracy when they are outputting language in a time-pressed automatic production.

Previous research studying learners of various L2 also showed that language learners faced difficulty in attaining production competence regarding some grammatical features, despite an advanced language proficiency level. Grüter et al. (2012) found that high-proficiency L2 Spanish learners showed difficulty in gender marking for nouns with real-time processing in an elicited production task. Siyanova and Schmitt (2008) reported that advanced Russian learners of English could not automatically produce English collocations with the same fluency as native speakers.

Our result that Chinese and Spanish EFL learners’ grammatical sensitivity significantly exceeded their production competence was also supported by a few previous studies on language perception and production. Linebaugh and Roche (2015) found that L2 learners’ accurate production is generally preceded by their L2 perception, even though production may enhance learners’ perception. Ahmadian (2012) supported L2 English learners of lower, intermediate, and even advanced proficiency showed problems producing accurate English articles, although they learned the grammar knowledge of articles well. The findings of the present study further confirm that EFL learners confront problems building some structures even though they are familiar with related grammar knowledge. Our study contributes additional evidence showing that advanced EFL learners’ language competence of production does not develop simultaneously with their grammatical knowledge. Targeting the problem of the unbalanced development of EFL learners’ language knowledge and language production competence, the following section will offer pedagogical implications for practitioners to improve learners’ production competence in the EFL contexts.

## Pedagogical implication

### Implicit knowledge fostering EFL context

**Figure 3:**
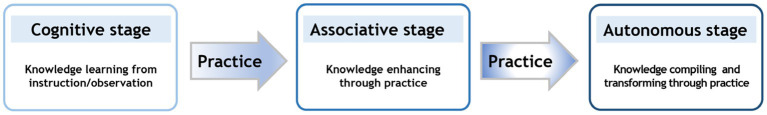
Berry (1987)‘s three-stage model of IK acquisition.

A primary concern for improving learners’ production competence is to promote EFL learners’ degree of IK, which, to be specific, lies in how to encourage the simultaneous development of learners’ language knowledge and production competence. It is closely related to how to convert the ‘monitoring’ function of grammar knowledge into the ‘driving’ power from subconscious grammar sensitivity. The conversion process highly conforms to the first type of IK in the seminal definition by Berry (1987), namely, the IK that was once explicit and declarative but gradually evolved into subconscious IK. Berry (1987) described a classical three-stage model of the IK formation process comprising a cognitive stage, an associative stage, and an autonomous stage (as shown in Figure 3). According to Berry’s (1987) model, implicit knowledge develops in three stages: the cognitive stage, where instruction or observation aids in knowledge acquisition; the associative stage, where practice helps to transform knowledge acquired in the previous stage into production; and the autonomous stage, where practice allows learners to process knowledge to the degree that they unconsciously produce what they have learned, at which point it becomes procedural or implicit. To gauge our EFL participants’ performance, we found that learners showed grammatical sensitivity but could not produce certain grammatical structures correctly. This suggests that in their learning process, learners were still in the cognitive stage and had only learned some grammatical rules of the structure, and they had not yet reached the associative and autonomous stages. To ‘implicitize’ the knowledge from its declarative and explicit predecessor, it is crucial to assist language learners in moving from the cognitive stage to the associative stage, where they can use the language structures in correct forms. And it is important to foster learners’ IK acquisition when moving from the associative stage to the autonomous stage in which they can produce language ‘without thinking’. From Figure 3, we can see that in the two transitions of the in-between stage, a key word ‘practice’ is mentioned. To put it in the domain of L2 acquisition, ‘practice’ does not mean, in no way, to do mechanical drills and repetitions to promote learners’ acquisition of IK. Instead, we consider that it will be effective if ‘practice’ is conducted in production-oriented learning activities based on meaningful interactions in communicative episodes.

### Production-oriented and interaction-based EFL pedagogical principles

In EFL environments, classroom-based language teaching and learning contexts are prevailing. Therefore, pedagogical concepts must assist teaching designers or practitioners to “foster acquisition-rich interaction” (Ellis, 2017). To reduce the discrepancy between grammar knowledge and language production identified in our study among EFL learners, we propose constructing language class activities that are production-oriented (Wen, 2018) and interaction-based (Ellis, 2017; Oliver et al., 2017; Adams and Oliver, 2019). The following subsections provide more details on the concepts we put forward.

### Production-oriented approach tasks

In EFL contexts, one of the most prominent challenges teachers and learners must face is the lack of natural communicative contexts for learners to practice the L2. We suggest that creating communicative contexts in the teaching process focusing on output be conducive for learners to produce an L2. Meanwhile, how to encourage learners’ willingness to produce the target language structures is also a pedagogical concern that practitioners need to consider. Wen’s (2018) *Production-oriented Approach* (POA) tasks offer teaching guidelines to solve the problem of insufficient communication and production in EFL contexts. In POA, the first stage, namely the motivating stage, aims at getting learners prepared to participate in activities and notice their gaps in knowledge and competence (He and Oltra-Massuet, 2021). Language learners are encouraged to recognize their own deficiencies in language knowledge. During this process, they may resort to their explicit language knowledge, which serves as the basis for their subsequent production. In the second stage of POA, enabling stage, learners are provided contexts for them to conduct specific mini-production tasks in close-to-life communicative scenarios. The target structures are used in communicative activities when they are working on completing their production tasks. Therefore, their explicit knowledge is covertly or explicitly practiced, which helps to enhance their comprehension of knowledge and automaticity of production. The final assessing stage includes teacher feedback, peer feedback, and learners’ self-evaluation on learners’ production, which consolidates learners’ knowledge and confirms their achievement in language production.

### Interaction-based production-oriented approach tasks

Interaction should be taken into full consideration in POA tasks, as interaction fosters EFL learners’ implicit learning of target language structures. Interaction promotes learners’ L2 acquisition in that interactive activities, such as implicit and explicit multi-source feedback, provide learners with opportunities to recognize problems in their interlanguage and propel them to produce modified output (VanPatten and Williams, 2015). In reviewing POA, Ellis (2017) also suggested that the inclusion of interactive activities into POA facilitates learners’ acquisition of target language structures. Therefore, interactive activities should be incorporated into designing the whole production-oriented tasks.

According to Gass and Mackey (2015), three main components of the interaction approach that account for the learning process are exposure to the target language, production of the language, and feedback on the production, which links learners’ language acquisition with the cognitive mechanisms of noticing, working memory, attention, and automation. These essential concepts are represented in interactive activities such as elaboration, recasting, repetition, or feedback. Merging these interactive activities into the three-stage procedures of each POA mini-task boosts its interactive function and facilitates learners’ language acquisition.

**Figure 4:**
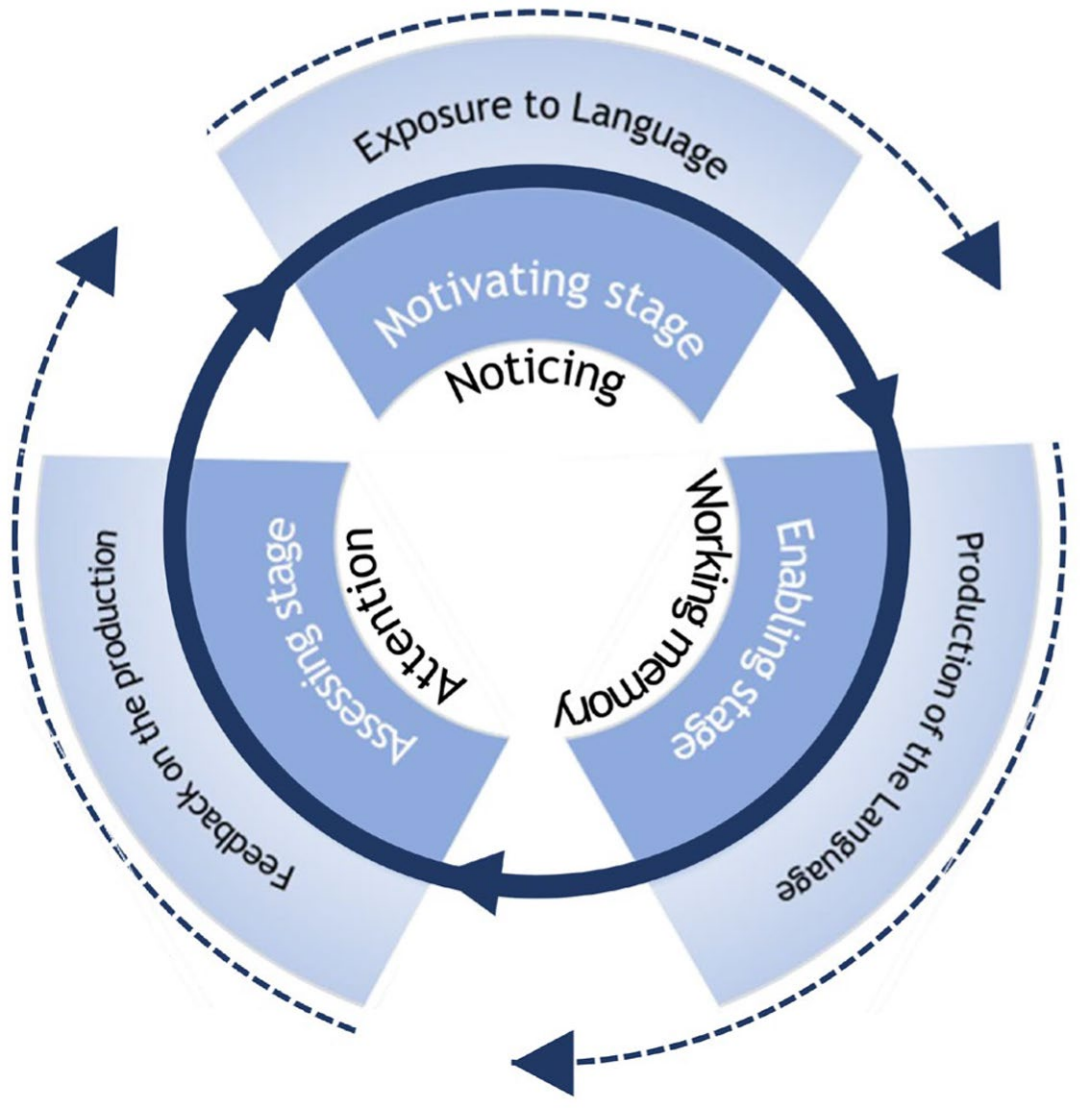
Interaction-based POA tasks.

As shown in Figure 4, the psychological mechanism that includes noticing, working memory, and attention links interaction and language learning, which is at the core of the Interaction-based POA tasks. These core concepts are then realized in mini tasks designed for the three POA stages for promoting learners’ language production in communication. Finally, interactive activities such as exposure to language, production of the language, and feedback on the production are carried out throughout the three POA stages. The whole Interaction-based POA tasks can be cycled throughout the whole learning process until learners automatically produce the target language, stimulating them to acquire target structures with communicative motives in the simulated scenarios. The cycling process explains Berry’s (1987) key element of ‘practice’ in an interactive way rather than using mechanical drills in forming the IK. The entire interaction process loaded on POA tasks pushes language learners to advance from the cognitive stage to the associative stage and finally reach the autonomous stage, facilitating their acquisition of IK.

## Conclusion

In the present study, we adopted the OEIT test to measure learners’ grammatical sensitivity and language production competence on English questions with refined grammatical errors. Chinese and Spanish EFL participants’ performance was analysed compared to native speakers, to probe the degree of implicit language knowledge acquired by EFL learners from two different L1s. The OEIT data was dissociated to study their grammatical sensitivity and production competence, revealing their development trajectory of language production competence. In sum, this study showed that: (1) both Chinese and Spanish EFL learners face great difficulty in acquiring IK of English questions, despite having attained an advanced proficiency level; (2) Chinese and Spanish EFL learners’ grammar knowledge and production competence do not develop simultaneously, and their production competence falls behind their level of grammar knowledge. Our results also support previous studies (Siyanova and Schmitt, 2008; Ahmadian, 2012; Grüter et al., 2012; Linebaugh and Roche, 2015) showing that even advanced language learners still confront difficulty in acquiring high production competence in certain grammatical features. The findings answered the question raised in He and Oltra-Massuet (2021) that certain types of errors, such as choice of auxiliaries (their GAUXC), produced by preliminary learners in English question formation, persist in the production from advanced EFL learners.

Based on the findings of this study, pedagogical implications have been formulated following Berry’s (1987) three-stage model of developing IK that derives from explicit and declarative knowledge, developed within Wen’s (2018) POA. We suggest that Interaction-based POA tasks assist EFL learners in attaining balanced development of their language knowledge and production competence, promoting their IK acquisition.

However, the present study is not without limitations. First, although our sample size is based on similar sizes in related studies, larger and more extensive participants would be preferable for more reliable results in future studies. Second, the present study focused only on implicit knowledge measurement, including experiments for directly testing participants’ grammatical knowledge would add additional support for exploring the development of language knowledge and production competence.

More research is needed along both theoretical and practical inquiries to support the findings of the present study. First, a series of experiments, including written tests, explicit language knowledge measurements, and IK measurements, need to be performed to explore the evolving mechanism of EFL learners’ language competence development. Second, action research based on the pedagogical implications discussed above needs to be conducted to trace the practical value of the findings achieved in this study so as to generate more theoretical and practical contributions to the language teaching and research field.

## Data availability statement

The raw data supporting the conclusions of this article will be made available by the authors, without undue reservation.

## Ethics statement

The studies involving human participants were reviewed and approved by Institutional Review Board of Universitat Rovira i Virgili (Approval code: CEIPSA-2021-TD-0002). The patients/participants provided their written informed consent to participate in this study.

## Author contributions

QH conceptualized the topic, designed and conducted the experiment, analyzed the data and wrote the manuscript. IO-M conceptualized the topic, instructed the experiment, and reviewed and proof-read the manuscript. All authors contributed to the article and approved the submitted version.

## Funding

This research has been partially supported by the URV Doctoral Program in Humanistic Studies (QH), grant 2021PFR-URV-72 (URV Program for Fostering Research) and the Serra Húnter Program (Catalan Government) (IO-M).

## Conflict of interest

The authors declare that the research was conducted in the absence of any commercial or financial relationships that could be construed as a potential conflict of interest.

## Publisher’s note

All claims expressed in this article are solely those of the authors and do not necessarily represent those of their affiliated organizations, or those of the publisher, the editors and the reviewers. Any product that may be evaluated in this article, or claim that may be made by its manufacturer, is not guaranteed or endorsed by the publisher.

## Footnotes

^1^www.Gorilla.sc
