# Supplementary material for: An experimental study on grammatical sensitivity and production competence in Chinese and Spanish EFL learners and its implications on EFL teaching methods
Source: Front Psychol. 2023 Feb 21;14:1096875. doi: 10.3389/fpsyg.2023.1096875 (PMC9989291; doi:10.3389/fpsyg.2023.1096875)
Supplement: Supplementary file 1 [file Data_Sheet_1.PDF]

Appendix A

Demographic information of participants

	NS group (%)	CS group (%)	SS group (%)
Age			
18-20	1 (8.3)	1 (3.1)	5 (13.5)
21-30	3 (25)	30 (93.8)	25 (67.6)
31-40	8 (66.7)	1 (3.1)	7 (18.9)
Gender			
Male	4 (33.3)	10(31.2)	20 (54.1)
Female	8 (66.7)	22 (68.8)	17 (45.9)
Education			
Secondary school	1 (8.3)	0 (0.0)	4 (10.8)
Undergraduate	8 (66.7)	15 (46.9)	10 (27.0)
Graduate	3 (25)	17 (53.2)	23 (62.2)
Starting year			
Kindergarten	NA	0 (0.0)	7 (18.9)
Primary school	NA	25 (78.1)	24 (64.9)
Junior secondary school	NA	7 (21.9)	4 (10.8)
University	NA	0 (0.0)	2 (5.4)
Residence in an English-speaking country			
Yes	NA	0 (0.0)	6 (16.2)
No	NA	32 (100.0)	31 (83.8)

Appendix B

The scoring criteria in terms of sensitivity and production score

1. If participants produced the original ungrammatical sentences they heard, or the wrong sentence type, such as a WH-question for a Y/N question, or failed to produce the sentence, the score was zero. For example, if participants were required to imitatively produce the question ‘Did your uncle visit you yesterday?’, they produced the question ‘Why did your uncle visit you yesterday?’, or they did not produce any word at all, the score of sensitivity and production was zero for this item.

2. A sensitivity score was given if participants intended to modify the ungrammatical sentence but did not produce a proper sentence (e.g., using the wrong tense, person or word order). For example, when participants heard the sentence ‘*Does John and Mary go to the library yesterday?’, they were supposed to say ‘Did John and Mary go to the library yesterday?’. If they produced the sentence ‘*Do John and Mary go to the library yesterday?’, a sensitivity score was identified, but the production score was zero because they did realize the grammatical problem in subject-verb agreement, but they did not produce it in the correct form required by the past tense indicated by the adverbial ‘yesterday’.

3. The following cases were all counted into successful production. One score was given in both sensitivity and production count if (a) participants modified the ungrammatical sentence and produced it in the grammatical form; (b) participants produced a correct question using a different expression for the same meaning; or (c) participants corrected a morpho-syntactic feature in an ungrammatical sentence with

mistakes not influencing the structure under analysis, e.g., using a wrong article or preposition, or changing a proper name; the sentence was also counted as correct production.
